# Exploring surface‐guided systems for intelligent breathing‐adapted four‐dimensional computed tomography: A comparison to infrared‐based reflective marker systems

**DOI:** 10.1002/acm2.70054

**Published:** 2025-02-24

**Authors:** Niklas Lackner, Andre Karius, Rainer Fietkau, Christoph Bert, Juliane Szkitsak

**Affiliations:** ^1^ Department of Radiation Oncology Universitätsklinikum Erlangen Friedrich‐Alexander‐Universität Erlangen‐Nürnberg Universitätsstraße 27 Erlangen Germany; ^2^ Comprehensive Cancer Center Erlangen‐EMN (CCC ER‐EMN) Erlangen Germany

**Keywords:** 4DCT, respiratory motion, surrogate system, technical feasibility study

## Abstract

**Purpose:**

This study evaluates the technical feasibility of adapting a surface monitoring system, designed for conventional four‐dimensional computed tomography (4DCT), to an intelligent, breathing‐adapted 4DCT and examines its potential to expand the currently limited range of supported surrogate systems.

**Methods:**

In an experimental phantom setting, we compared breathing curve quality and its impact on breathing‐adapted 4DCT generation between a surface monitoring camera and our clinical infrared (IR) system, using a research‐grade IR camera coupled with a radiation detector as an independent reference. Breathing curves from the surface monitoring system and the research‐grade camera were corrected for table motion. We assessed the influence of differences in breathing curves on the automatic selection of parameters before scanning, intelligent X‐ray triggering during acquisition, and the differences of binning point selection for reconstruction as well as image quality. Additionally, we simulated the impact of latency on image quality and measured the observed latencies between the surrogate systems relative to an X‐ray measurement.

**Results:**

During table movement, discrepancies were found in breathing signals from the surface monitoring system compared to the clinical and reference systems. After correcting for table motion, the surface monitoring system's curves aligned consistently with those of the other systems with amplitude (AMP) variations of less than 10% and breathing rate (BR) variations of less than 1%. Corrected curves showed improved performance in their ability to generate breathing‐adapted 4DCTs. The clinical IR system showed a 45 ms latency advantage over the surface monitoring system, impacting image quality as simulated.

**Conclusions:**

After correcting surface monitoring breathing curves, satisfactory agreement with the clinical and independent reference systems was achieved. With modifications, the surface monitor solution could serve as a suitable surrogate for breathing‐adapted 4DCT. In our experimental setting, the surface monitoring system had a 45 ms delay relative to the clinical system, potentially affecting image quality.

## 1 INTRODUCTION

Radiotherapy (RT) for cancer of the thorax and upper abdomen often relies on utilizing respiration‐correlated four‐dimensional computed tomography (4DCT) to localize tumors affected by breathing motions. Conventional 4DCT algorithms produce time‐resolved images of the patient's anatomy at different stages of the breathing cycle by synchronously gathering X‐ray projections and respiratory signals, where they retrospectively assign projection data to specific breathing phases for image reconstruction.^1^

In contrast to this conventional 4DCT method,^2^, ^3^, ^4^, ^5^ an early suggestion was made to utilize real‐time analysis of breathing signals to control projection data acquisition.^6^, ^7^ Recently, a combined approach was introduced, the so‐called intelligent 4DCT (i4DCT) algorithm and scan mode. This method uses real‐time prospective scan triggering using an online analysis, tailoring the scanning process to each patient's specific breathing pattern and optimized retrospective selection of projection data.^8^, ^9^ Studies involving *in‐silico* simulations, quality assurance measurements, phantom and patient data have shown that i4DCT significantly reduces image artifacts caused by irregular breathing, compared to conventional spiral 4DCT.^9^, ^10^, ^11^, ^12^

While conventional 4DCT supports a large number of different surrogate systems,^13^, ^14^, ^15^, ^16^ i4DCT presents challenges due to its stringent demands, requiring high quality breathing data and real‐time surrogate data transmission. Currently, the i4DCT approach^17^ supports only two systems: one using infrared (IR) marker tracking^15^ and the other utilizing a belt pressure sensor.^18^, ^19^ For the belt pressure sensor system, a recent study has shown that the quality of the recorded breathing curve can affect image quality in conventional 4DCT imaging.^19^

The aim of this paper was to assess whether the surface camera system could serve as an adequate alternative to the clinically used IR system for creating breathing‐adapted 4DCTs, or if it could be adapted, with or without modifications, for broader applications beyond its current clinical use. A second IR camera system served as an independent reference. Our examinations included an analysis of the breathing curves, which for non‐table‐mounted camera systems were corrected to account for table motion. We investigated the impact of the different breathing curves on automatic parameter selection before scanning, triggering the X‐ray‐on/‐off signal and the selection of binning points during reconstruction. Additionally, the effect of latency on image quality was assessed in simulations and latency was measured between the different camera systems.

## 2 METHODS

### 2.1 i4DCT scanning

A SOMATOM go.Open Pro multi‐slice CT scanner (Siemens Healthineers AG, Forchheim, Germany) was used. 4DCT scans were acquired using the i4DCT algorithm with sequential scanning at 120 kVp, 64 × 0.6 mm collimation, and a fixed table feed of 0.9 × 64 × 0.6 mm, with automated tube‐current modulation to optimize dose. To streamline workflow and minimize manual errors, Fast 4D automatically selects scan parameters, such as gantry rotation time, based on respiratory curve analysis performed in the training region prior to the scan. During data acquisition, the i4DCT algorithm adjusts its ‐ray‐on/‐off selection based on real‐time analysis. By intelligently adjusting the reconstruction bins to align with the breathing phase, aiming to closely match it with the reference breathing curve (RBC), binning point selection is optimized.^20^ Details about Fast 4D and i4DCT can be found in previous publications.^8^, ^9^ Reconstructions of 10 amplitude (AMP)‐ and 10 phase‐based images were performed using the nonclinical reconstruction software “ReconCT” (v14.2; Siemens Healthineers AG, Forchheim, Germany). The software allows a retrospective exchange of breathing curves and reconstruction of CT projection data with these curves.

### 2.2 Surrogate systems

**FIGURE 1:**
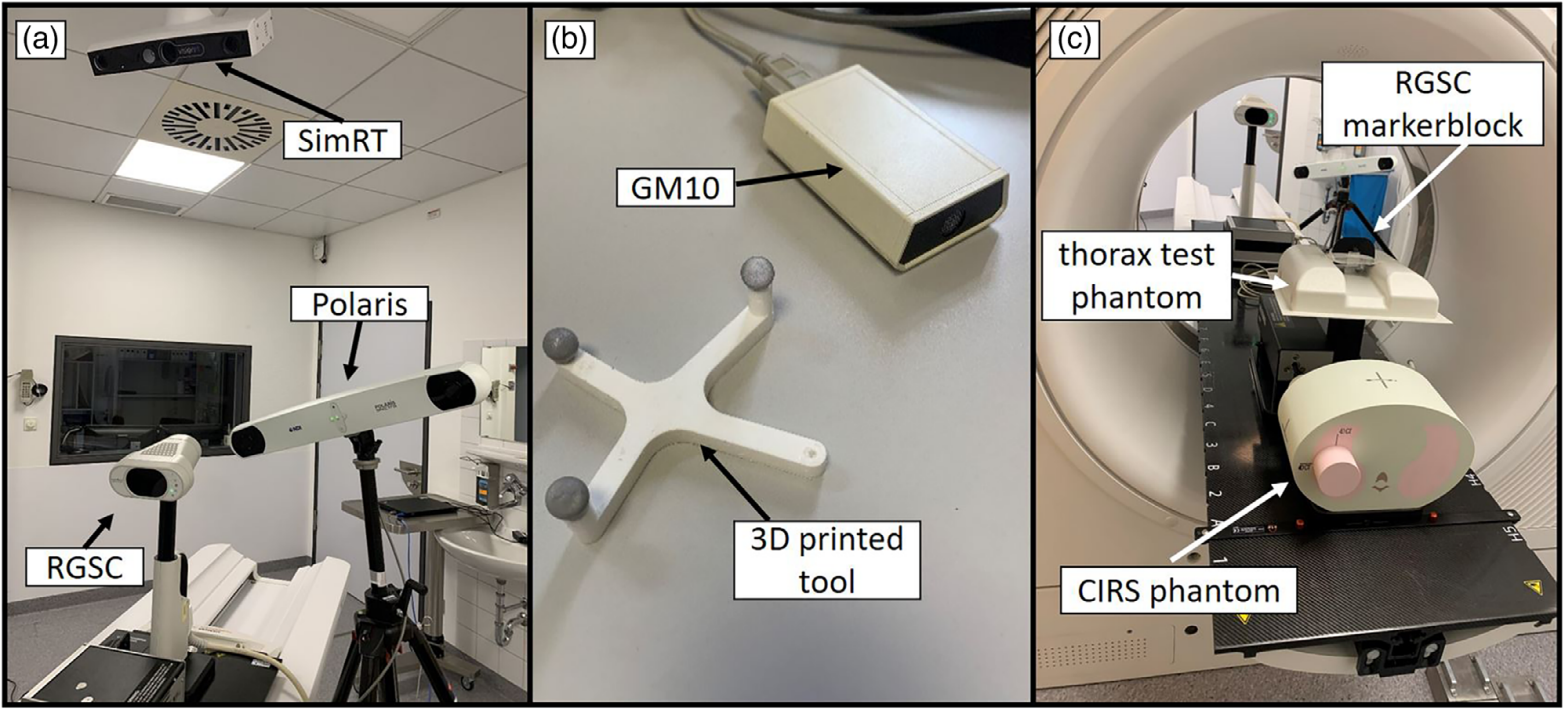
Measurement setup for the different surrogate systems. (a) Camera systems used in the experimental setup. (b) Geiger Müller Counter (GM10) and the 3D printed tool. (c) Phantoms used are shown from a behind‐the‐bore view.

Three optical surrogate systems were used in this study (see Figure 1a): the Respiratory Gating for Scanners system (RGSC; version 1.1.25.0, Varian Medical Systems, USA), SimRT (version 7.2, VisionRT, UK), and a Polaris Spectra (Northern Digital Inc., Canada).

The clinically used RGSC system consists of a table‐mounted IR camera. It uses reflections from a passive marker block placed on the patient's chest to determine the breathing curve. The breathing signal is transferred to the scanner in real‐time and used as input for the i4DCT algorithm.

SimRT focuses on monitoring the patient's surface and utilizes a ceiling‐mounted pod. Position variations are calculated using active stereo photogrammetry and triangulation within a 5 × 5 cm^2^ field of view (in SimRT‐specific terms: patch), which can be positioned individually on the patient's surface. SimRT's table movement correction was set to a fixed 34.5 mm table increment, consistent with the fixed increment of the i4DCT scanning protocol for 4DCT sequence scanning, which is necessary to propagate the breathing patch to different positions. It is important to note that the clinical SimRT installation used in this study was not originally designed for this particular use case in its current form. Therefore, SimRT was operated in an experimental setting to explore its potential application in this scenario.

The Polaris was used, in combination with a tripod and coupled to a Geiger Müller counter GM10 (Black Cat Systems, USA; see Figure 1b), to determine the ‐ray‐on status. The X‐ray‐on was matched with Polaris breathing curves. The camera system is similar to the RGSC system and is equipped with a positional sensor designed to determine the location of IR markers, which excels in tracking multiple points with 0.06 mm precision (Root Mean Square Error) and 16.6 ± 1 ms latency.^21^ NDI's 6D Architect (v3.02.04, Northern Digital Inc., Canada) was used for tool definition, enabling the system to track both the RGSC marker block and a custom 3D printed object equipped with IR markers (see Figure 1b). This high precision and low latency, combined with its ability to track multiple tools simultaneously, provide table movement information, and measure X‐ray‐on status, make it well suited as an independent reference.

### 2.3 Phantom measurements

We employed the dynamic thorax phantom model 008A (Computerized Imaging Reference Systems (CIRS), USA) for phantom measurements. This device mimics the dimensions of a human thorax, with a spherical movable tumor inside, and offers precise motion accuracy and reproducibility within ± 0.1 mm.^22^ To render the breathing motion of the phantom detectable by our surface systems, the “thorax test phantom” (VisionRT, UK) with the marker block attached to it was positioned atop the moving surrogate platform of the CIRS phantom, as depicted in Figure 1c. The 3D printed tool was also placed on the table, at the same table position as the RGSC marker block.

Basic sinusoidal curves were generated

(1) $$\zeta \left(t\right)=\frac{A_{p2p}}{2}\;\sin\left(\frac{2\pi t}{T_{cycle}}\right),$$

to understand complex interactions in i4DCT, focusing on RBC creation, binning placement, and latency effects. Various breathing signals ζ with peak‐to‐peak amplitudes from 2 mm ≤ $A_{p2p}$ ≤ 20 mm and periods ranging from 6 ≤ $T_{cycle}$ ≤ 20 breaths per minute (BPM) were selected to represent diverse breathing patterns reported in previous studies.^23^, ^24^

Given the difficulties presented by respiratory irregularities in 4DCT imaging, we also evaluated regular cos^6^ breathing curves

(2) $$\zeta \left(t\right)=\frac{A_{p2p}}{2}\cos^{6}\left(\frac{2\pi t}{T_{cycle}}\right)\;\;$$

and challenging cos^6^ breathing curves involving variations in AMP and frequency,^12^ drawing upon a comprehensive approach to encapsulate the complexity encountered in clinical settings.^24^

### 2.4 Data analysis

Breathing curves were exported for RGSC and SimRT, containing acquisition time, AMP values, and X‐ray‐on/‐off status. For the data acquisition with Polaris, we used Python 3.7.14 and the open‐source toolkit SciKit‐Surgery (https://github.com/SciKit‐Surgery/scikit‐surgerynditracker), along with the GM10 for X‐ray data. Data analysis was conducted using MATLAB (version R2019b, The MathWorks Inc., Massachusetts, USA). To match the breathing curves, the X‐ray‐on signal from the different systems was used, breathing curves were resampled to a 20 ms time grid resolution, and all curves were cut to the same length.

### 2.5 Breathing curve evaluation

**FIGURE 2:**
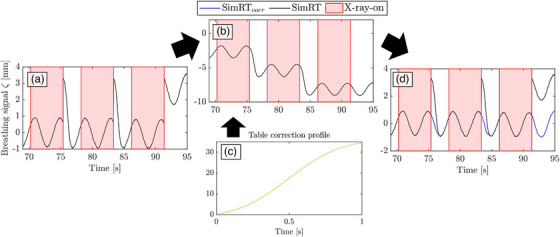
Retrospective fix for SimRT breathing curves. (a) Uncorrected SimRT breathing signal with peak artifacts caused by SimRT's patch propagation. (b) Removal of SimRT's correction for table movement and amplitude stability in 4DCT sequence scanning mode. (c) The table correction profile was measured using a combination of Polaris and the multi‐tracking approach. (d) Corrected (blue) and uncorrected (black) breathing signals.

In contrast to the RGSC system, both the Polaris and SimRT surrogate systems are not table‐mounted, which necessitates table movement corrections. The SimRT camera is ceiling‐mounted in its clinical configuration, while the Polaris system is positioned next to the patient couch, resulting in a different angle of view and potentially affecting its field of coverage and sensitivity to motion. To cancel out table motion when using the Polaris, both table and phantom motions were tracked with a 3D printed tool positioned (see Figure 1b) on the table alongside the RGSC marker block. The Polaris tracked both markers to subtract table motion from phantom motion. SimRT's clinical software corrects for table motion by moving the patch from one defined position to the next, after data acquisition at a defined table position is completed. Due to these system‐specific corrections, which were not in the intended scope of the clinical version of SimRT, artifacts occur as peaks in the breathing curve (see Figure 2a, details in Appendix Figure A). To correct the breathing curves, system‐specific corrections had to be removed (see Figure 2b). Additionally, for each peak, a scaling factor is derived by comparing the height of the system‐specific peak artifact to the maximum of the breathing curve immediately preceding it. This scaling factor in SimRT is likely needed for AMP stability, as the patch travels deeper into the CT scanner. After removing the SimRT corrections, the table motion profile (see Figure 2c), measured with the Polaris, was multiplied by the derived scaling factor and is successively added to the region after the X‐ray‐on to X‐ray‐off (Figure 2d). To demonstrate that our correction is an improvement compared to the unprocessed SimRT breathing curves we included both the uncorrected SimRT and the corrected SimRT_corrected_ curves in the i4DCT analysis in subsequent sections.

For the breathing curve evaluation, absolute values of AMP maxima for the different surrogate systems were compared. Subsequently, curves were normalized to the first local minimum after the first X‐ray‐on. AMP normalization of breathing curves is justified as the i4DCT algorithm utilizes relative AMPs of the curves. To temporally align the breathing curves of the different surrogates to the 4DCT raw data, curves were shifted to match, to correct for any latency between the systems. Breathing curves from 4DCT raw data were exported using ReconCT. Two representative cases were selected: a regular sin‐case and an irregular cos^6^‐case with variations in phase and AMP. We compared the breathing curves for the different surrogate systems in terms of estimated breathing rates (BRs) (min, max, median, and mean) as well as AMP and phase variations for the entire breathing curves. To estimate the BPM, the different signals were analyzed for individual breathing cycles. Inhalation peaks and exhalation valleys were identified using a peak detection algorithm, with each valley pair representing one complete cycle. The interval between the start of consecutive cycles was calculated and the mean BR was calculated by averaging the BPM across all cycles.

### 2.6 Automatic parameter selection and intelligent X‐ray selection

The Fast 4D algorithm performs automatic parameter selection by analyzing the patient's breathing pattern during a 20‐s training period prior to the scan, automatically adjusting key parameters such as gantry rotation time. Similarly, the i4DCT algorithm analyzes a 20‐s training period to determine when X‐ray‐on and X‐ray‐off events are allowed to trigger. Differences in automatic parameter selection between the two representative breathing curves were investigated by examining the training period and simulating X‐ray‐on and X‐ray‐off selection based on the i4DCT algorithm.^8^ These results were then compared across the different systems.

### 2.7 Influence of binning placement on image quality

For the two representative cases RBCs were created for each surrogate system. Based on these, optimized binning placements were calculated for 10 amplitude‐ and 10 phase‐based reconstructions.^8^ The simulated tumor was segmented in every breathing phase based on Hounsfield Unit (HU) thresholding to evaluate image quality in phantom measurements. The center of mass (COM) was determined for every tumor position and examined in inferior‐superior direction. The trajectories were normalized to maximum inspiration for AMP‐based reconstruction and to maximum expiration for phase‐based reconstruction. The COM trajectory for phase‐based reconstruction was inverted in AMP.

### 2.8 Impact of latency on image quality and the determination of system latencies

**FIGURE 3:**
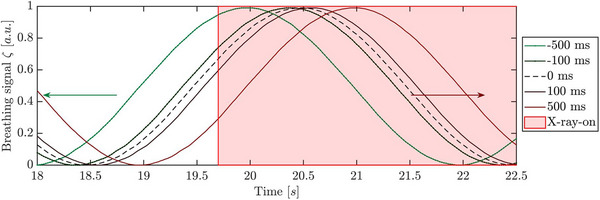
Artificially shifted breathing curves generated from a phantom measurement, later used for latency simulations. For clarity, only a few curves are shown. The green arrow indicates curves shifted ahead in time, while the red arrow indicates curves shifted to lag.

Latency simulations were performed to estimate the influence of latency on the image quality, particularly on the associated tumor position. Latency was artificially introduced in a simple sin‐case measurement by systematically shifting breathing curves within a predefined range. The shifts ranged from ‐100 to 100 ms, in increments of 20 ms (see Figure 3). Additionally, two more extreme shifts of −500 and 500 ms were included. To evaluate the impact of latency on image quality, images were reconstructed with the artificially generated breathing curves using the reconstruction software ReconCT (Section 2.1). The evaluation of image quality was performed according to Section 2.7.

Additionally, we carried out latency measurements to investigate the surrogate system‐specific delays. In the latency measurements, we used the set from Section 2.3. 4DCT raw data, RGSC, SimRT, and, as a reference, the Polaris, which was matched with the GM10, were compared. To estimate the latency between the different systems, breathing curves were extracted from the 4DCT raw data (“RGSC online”) using ReconCT as well as exported from RGSC (“RGSC offline”) and SimRT (“SimRT offline”). All the different signals were aligned to their respective first X‐ray‐on signals. To overcome noise, the curves were smoothed and fitted. The shift in maxima between the different curves after the first X‐ray‐on signal was calculated. Latencies were compared against the Polaris signal matched with the GM10, which is the measured X‐ray‐on in contrast to the other X‐ray‐on signals representing an electronic pulse of the scanner to trigger irradiation, which is sent to the surrogates and the X‐ray tube of the scanner. For further details regarding the timing of the TTL‐in and X‐ray‐on signals, please refer to the timeline provided in the Appendix Figure D.

### 2.9 Feasibility for patients

Patient data from two lung tumor patients was retrospectively analyzed to validate the phantom results in a clinical setup. Data was acquired using the RGSC and SimRT system, simultaneously. To allow SimRT to record breathing at the same position as the RGSC system, the marker block was covered with white tape to be visible for SimRT and the patch was placed on the marker block. We evaluated SimRT, SimRT_corrected_, and RGSC curves. Additionally, we simulated and evaluated an i4DCT X‐ray‐on trigger scenario for these patients.

## 3 RESULTS

### 3.1 Breathing curve evaluation

For the entire measurement set, we found that the SimRT_corrected_ curves were consistent with the Polaris and RGSC breathing curves. When comparing maximum AMPs of the phantom breathing curves to the Polaris signal, RGSC overestimated curves by 2.36 ± 0.92% (i.e., for a 10 mm breathing AMP ∼0.3 mm deviation), while SimRT_corrected_ curves underestimated the maxima by −6.44 ± 3.39% (i.e., for a 10 mm breathing AMP up to ∼1 mm deviation). After this comparison, curves were normalized, as mentioned in Section 2.3.

**FIGURE 4:**
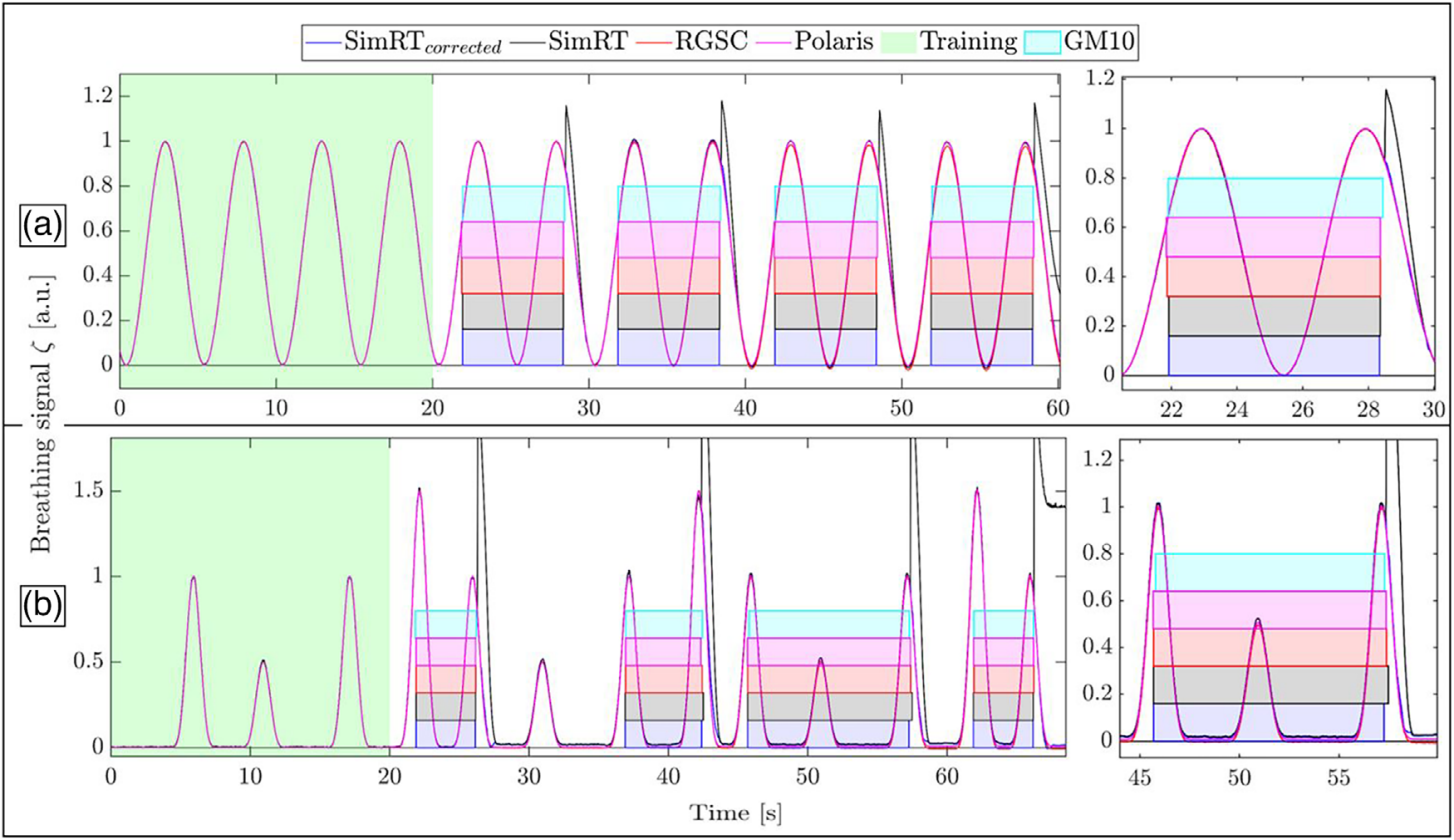
Two exemplary cases for the breathing curve comparison of the different surrogate systems and additionally measured and simulated X‐ray trigger selection in i4DCT for (a) a regular sin‐case and (b) an irregular cos^6^‐case, displaying the entire curve on the left and a zoomed region on the right. The green area is the training area, which covers at least three entire breathing cycles to generate a reference breathing curve. It is used as the foundation for phase space X‐ray trigger constraints and is updated each breathing cycle. The differently colored transparent fields show the simulated X‐ray triggers for the breathing curves of the different surrogate systems matching the colors of the breathing curves, while the transparent cyan field shows the measured X‐ray signal.

For the regular sin‐case curves (see Figure 4a), the data presented in Table 1a reveals a high level of consistency in the BR and AMP deviation for the training period across all surrogate systems. In the entire observation period, there was a noticeable increase in variability, especially in the SimRT system, which recorded a higher maximum BR at 14.02 BPM, substantially deviating from other systems where it remained around 12.10 BPM (RGSC, Polaris) to 12.15 BPM (SimRT_corrected_). The minimum BR also showed more variation, notably in SimRT, which dropped to 10.49 BPM compared to other systems, which maintained values close to 11.90 BPM. Additionally, SimRT showed increased variability, with a standard deviation (STD) of the BR at 1.39 BPM and a notably high STD of AMP at 10.30%, suggesting greater fluctuations in measurements compared to other systems.

**TABLE 1:** Breathing curve statistics for the breathing curves generated with the different surrogate systems for two exemplary cases (a) a regular sin‐case and (b) a challenging cos^6^‐case irregular in phase and amplitude (AMP). For the sin‐case, a stable breathing rate (BR) and low standard deviations (STD) are expected, whereas for the challenging cos^6^‐case, variations in BR and deviations in AMP are anticipated. In both cases, during the training period, all systems aligned well. Throughout the entire duration, Polaris, SimRT_corrected_, and RGSC matched closely, while SimRT showed differences.

	Training				Entire			
sin‐case	Polaris	SimRT_corrected_	SimRT	RGSC	Polaris	SimRT_corrected_	SimRT	RGSC
Max BR (BPM)	12.00	12.05	12.05	12.05	12.10	12.15	14.02	12.10
Min BR (BPM)	11.95	11.95	11.95	11.95	11.90	11.86	10.49	11.90
Mean BR (BPM)	11.98	12.00	12.00	12.01	12.00	12.00	12.02	11.99
Median BR (BPM)	12.00	12.00	12.00	12.05	12.00	12.05	11.98	12.00
STD BR (BPM)	0.02	0.05	0.05	0.06	0.05	0.10	1.39	0.06
STD AMP (%)	0.01	0.08	0.08	0.02	0.13	0.61	10.30	0.96

	Training				Entire			
cos^6^‐case	Polaris	SimRT_corrected_	SimRT	RGSC	Polaris	SimRT_corrected_	SimRT	RGSC
Max BR (BPM)	12	12	12	11.95	16.04	16.13	17.34	16.13
Min BR (BPM)	9.65	9.65	9.65	9.68	9.62	9.68	9.26	9.62
Mean BR (BPM)	10.70	10.70	10.70	10.70	12.00	12.00	12.00	11.99
Median BR (BPM)	10.70	10.70	10.70	10.70	12.00	11.91	12.05	12.00
STD BR (BPM)	1.66	1.66	1.66	1.61	2.25	2.25	2.23	2.25
STD AMP (%)	34.65	33.98	33.98	34.80	37.89	37.32	60.79	38.01

Table 1b details the BR statistics for the irregular cos^6^‐case (see Figure 4b), an irregular breathing curve in both phase and AMP, across various surrogate systems. Similar to the regular sin‐case the data revealed a high level of consistency in the BR deviations and STD AMP deviations during the training period across all surrogate systems. During the entire observation period, the uncorrected SimRT system recorded a maximum BR of 17.34 BPM and an STD AMP as high as 60.79%, emphasizing greater irregularity compared to the other surrogate systems, again likely related to the peak artifacts.

### 3.2 Automatic parameter selection and intelligent X‐ray selection

The values displayed in Table 1 indicate strong consistency across all surrogate systems during the training period of the breathing curves (see Figure 4a–b green regions), which consists of the interval 20 s before the first X‐ray activation. This uniformity suggests that the Fast 4D automatic protocol selection would be consistent when using input from different surrogate signals. As displayed in the previous section, within this training region, metrics such as maximum, minimum, mean, and median BR, along with their STDs and AMP STDs, show minimal variation across the different surrogate systems.

The simulated X‐ray‐on/‐off trigger, depicted in Figure 4a for a regular breathing curve and in Figure 4b for an irregular breathing curve, consistently maintained a synchronization pattern compared to the experimental X‐ray trigger measurement for all surrogate systems.

For the regular breathing curve, the mean difference in X‐ray trigger times was compared to the measured independent reference system. The differences were 37.5 ± 44.1 ms, 47.5 ± 31.5 ms, and 27.5 ± 31.5 ms for Polaris, RGSC, and SimRT, respectively, with SimRT_corrected_ showing a difference of 37.5 ± 38.0 ms. The measured independent reference system showed a mean X‐ray‐on time duration of 6.52 s ± 34.6 ms. The X‐ray‐on time durations for the simulated X‐rays were 6.555 s ± 45.6 ms, 6.495 s ± 21 ms, and 6.485 s ± 26 ms for Polaris, RGSC, and SimRT, respectively, with SimRT_corrected_ having a duration of 6.465 s ± 26 ms.

The mean differences in X‐ray trigger times for the irregular breathing curve, compared to the measured independent reference system, were 22 ± 68 ms, −2.5 ± 56.1 ms, and ‐10 ± 87 ms for Polaris, RGSC, and SimRT, respectively, with SimRT_corrected_ showing a difference of 37.5 ± 42.9 ms. The measured independent reference system, GM10, showed a mean X‐ray‐on time duration of 6.41 ± 2.98 s. For the simulated systems, the mean durations were 6.415 ± 3.09 s, 6.445 ± 3.07 s, and 6.49 ± 3.11 s for Polaris, RGSC, and SimRT, respectively, with SimRT_corrected_ having a duration of 6.395 ± 3.03 s.

We found that all the simulated phantom breathing curves would trigger X‐ray‐on/off in the same regions of the breathing curves. This indicates that the simulated systems closely matched the measured independent reference in terms of X‐ray‐on times with minor X‐ray trigger differences (< 100 ms) and small differences in mean X‐ray duration (< 100 ms) across the systems.

### 3.3 Influence of binning placement on image quality

**FIGURE 5:**
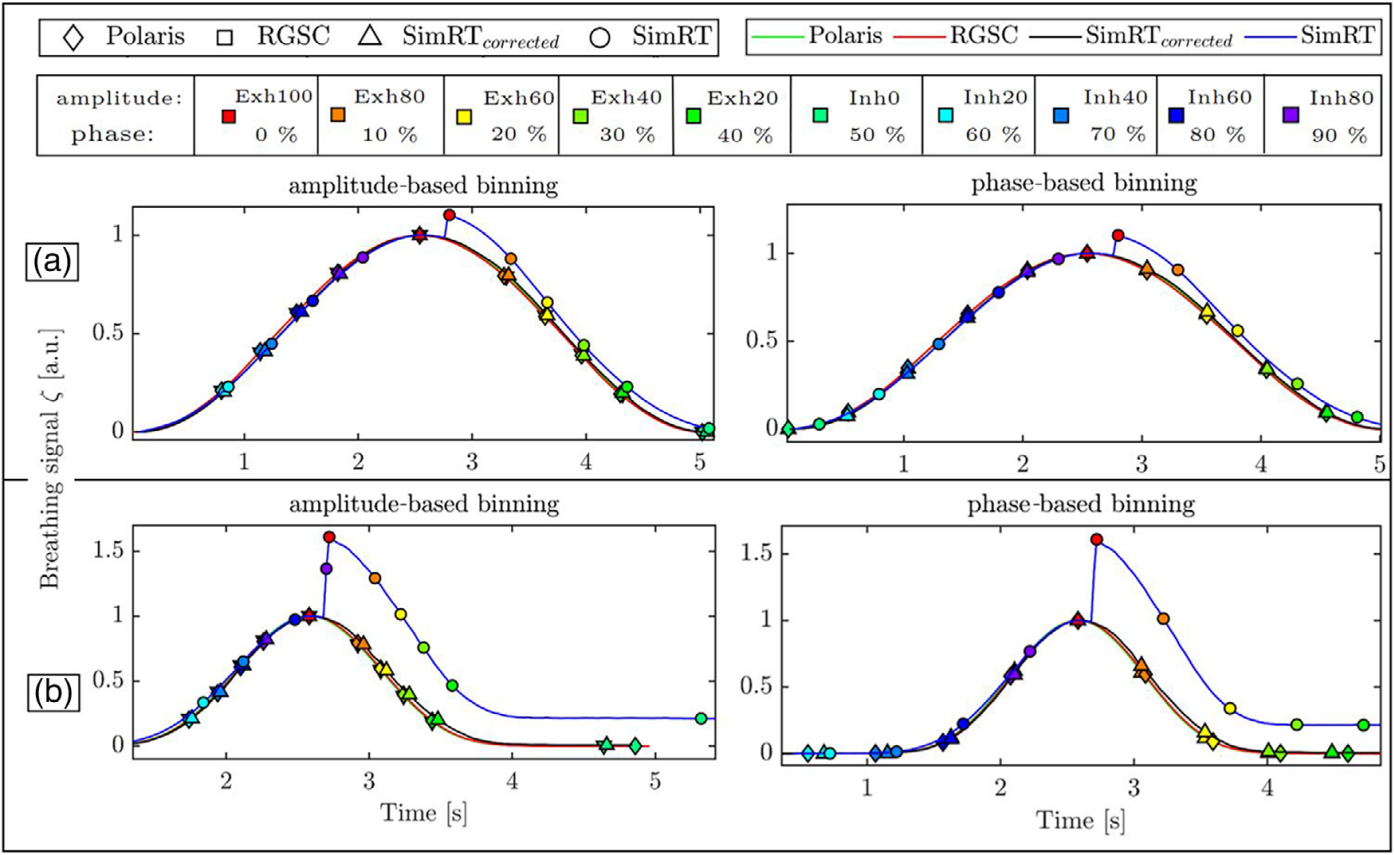
Reference breathing curves of the different surrogate systems for amplitude‐ and phase‐based binning for (a) a regular sin‐case and (b) an irregular cos^6^‐case. Amplitude‐based reconstruction nomenclature: Inh0 to Inh80 for inhalation and Exh20 to Exh100 for exhalation. For phase‐based reconstruction, percentages from 0% (start of exhalation) to 90% (end of inhalation) are used. The reference breathing curves of Polaris, RGSC, and SimRT_corrected_ align closely, while SimRT's peak artifacts significantly distort its curve, particularly during exhalation.

Figure 5 shows the different RBCs for a regular sin‐case and an irregular cos^6^‐case, acquired with the different surrogate systems and the respective binning points. The peak artifacts included in the uncorrected SimRT signal were pronounced in both the RBC and the phase space representation of it, which can be found in the Appendix Figure B/C.

When comparing to the binning point timestamps of the Polaris in the regular breathing curve, the time points of AMP‐based binning of the RGSC differed by 4 ± 8 ms, SimRT_corrected_ by −22 ± 14 ms, and uncorrected SimRT by −96 ± 80 ms with higher deviations in the inhalation part (Inh20, Inh40, Inh60, Inh80, Exh100). For phase‐based reconstruction, binning points of RGSC matched Polaris exactly, while SimRT_corrected_ differed by 0.5 ± 5.1 ms, and uncorrected SimRT points were delayed strongly for all points by 260 ms.

In the irregular breathing curve, AMP‐based binning points were delayed compared to Polaris by 22 ± 66 ms for RGSC, 26 ± 12 ms for SimRT_corrected_, and 246 ± 181 ms for uncorrected SimRT, again with higher deviations in the inhalation part (Inh20, Inh40, Inh60, Inh80, Exh100). In phase‐based binning, RGSC was ahead by 15 ± 86 ms, SimRT_corrected_ bins matched Polaris exactly, and uncorrected SimRT points were delayed by 122 ± 72 ms. Overall, there was good agreement for both representative breathing curves in binning point values between Polaris, RGSC, and SimRT_corrected_, while SimRT values were affected by the deformed RBC due to the peak artifacts.

The RBCs significantly influence image creation. Each breathing cycle, which matches an X‐ray‐on cycle with its projection data, is compared to the RBC. This comparison determines the binning placement within the image stack, guiding the image reconstruction process.

**FIGURE 6:**
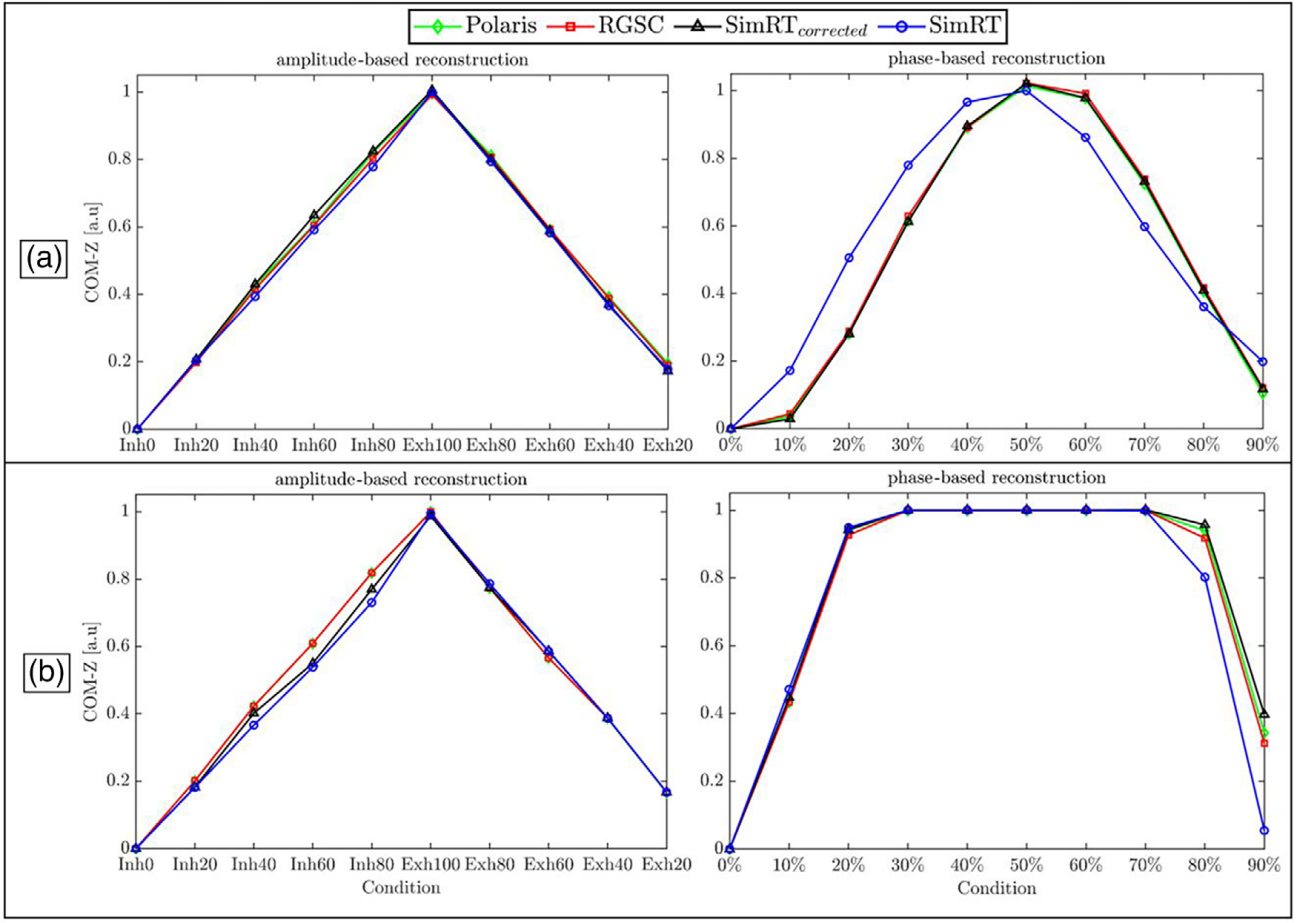
Results of the center of mass evaluation in inferior‐superior direction for the different surrogate systems using amplitude‐ and phase‐based reconstruction in (a) a regular sin‐case and (b) an irregular cos^6^‐case. Amplitude‐based reconstruction nomenclature: Inh0 to Inh80 for inhalation and Exh20 to Exh100 for exhalation. For phase‐based reconstruction, percentages from 0% (start of exhalation) to 90% (end of inhalation) are used. SimRT_corrected_, RGSC, and Polaris demonstrated good agreement, whereas SimRT showed a notable discrepancy, in particular for the phase‐based reconstruction.

Figure 6b shows the results of the AMP‐ and phase‐based reconstruction and the corresponding COM variations of the tumor insert along the inferior‐superior direction for the different surrogate systems. For the regular sin‐case, we compared against images created with breathing data from the Polaris for AMP‐based reconstruction and we found COMs in inferior‐superior direction differed for image data for RGSC by 0.1 ± 0.1 mm, for SimRT_corrected_ by −0.1 ± 0.2 mm and for SimRT by 0.2 ± 0.1 mm, indicating good agreement for all surrogate systems. In phase‐based reconstruction we found that compared to image data by Polaris, RGSC differed by −0.1 ± 0.1 mm, SimRT_corrected_ by 0.1 ± 0.1 mm and for SimRT higher differences of −0.4 ± 1.2 mm.

For the irregular cos^6^‐case, we again compared against images created with breathing data from the Polaris for AMP‐based reconstruction and we found COMs in inferior‐superior had excellent agreement with RGSC data and differed for SimRT_corrected_ by 0.1 ± 0.2 mm and for SimRT by 0.2 ± 0.4 mm indicating higher deviation than for the regular case were found, in particular in the inhalation part. In phase‐based reconstruction, we found that compared to image data by Polaris, RGSC differed by −0.1 ± 0.1 mm, SimRT_corrected_ by −0.2 ± 0.3 and again for SimRT higher differences of 0.5 ± 1.2 mm.

### 3.4 Impact of latency on image quality and the determination of system latencies

**FIGURE 7:**
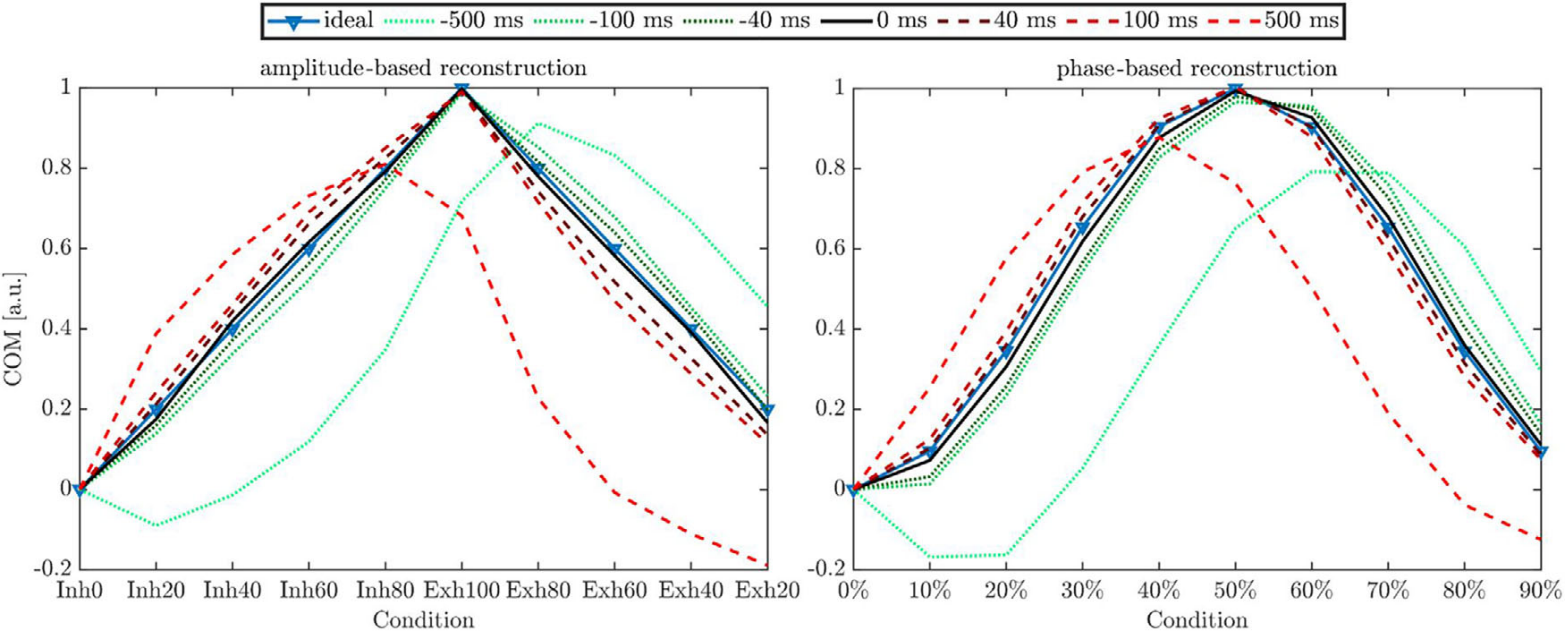
Center of mass (COM) differences for the simulation of the time‐shifted breathing curves visualised for amplitude‐based and phase‐based reconstruction—curves were normalized to the maximal COM position. In both plots, the red dashed lines show the COM results for the lagging curves, while the green dash‐dotted lines show the COM results for the curves shifted forward in time. Amplitude‐based reconstruction nomenclature: Inh0–Inh80 for inhalation and Exh20–Exh100 for exhalation. For phase‐based reconstruction, percentages from 0% (start of exhalation) to 90% (end of inhalation) are used.

Figure 7 presents the outcomes for the simulations and the determination of the tumor insert COMs in inferior‐superior direction resulting from the AMP‐ and phase‐based image reconstructions for the artificially latency shifted breathing curves. Notable is the asymmetry we found for the ideal mathematically calculated reconstruction for AMP‐based reconstruction and phase‐based reconstruction. In AMP‐based reconstruction the ideal COM trajectory was located between the reconstructions of the breathing curves 0 and −40 ms while for phase‐based reconstruction the ideal COM trajectory is located between the reconstruction for 0 and 40 ms latency, curve shift. Table 2 summarizes the mean differences and STDs for tumor COM estimations, highlighting the sensitivity of image quality to latency shifts.

**TABLE 2:** Statistics for the simulations of latencies, mean deviation values and standard deviations (STD) for the tumor center of mass, created from shifted breathing curves ranging from −500 to 500 ms. The non‐shifted reference curve is highlighted in green.

Latency results		−500 ms	−100 ms	−60 ms	−20 ms	0 ms	20 ms	60 ms	100 ms	500 ms
phase‐based	Mean (mm)	−1.73	−0.03	−0.04	−0.02	0	0.02	0.04	0.04	−1.15
	STD (mm)	2.84	0.60	0.25	0.12	0	0.12	0.38	0.63	2.63
amplitude‐ based	Mean (mm)	−0.97	0.03	0.03	0.04	0	−0.03	−0.06	−0.10	−1.80
	STD (mm)	3.05	0.65	0.37	0.12	0	0.13	0.39	0.67	2.99

The RGSC offline signal had the shortest delay, with a lag of −108.3 ± 10.3 ms, followed by the SimRT offline signal with a lag of −62.5 ± 27.34 ms. The online RGSC signal of the 4DCT raw data showed a delay of −17.5 ± 12.1 ms. A negative lag indicates that these systems were ahead of the measurements. All systems were compared to our real‐time measurements of the X‐ray signal using the Polaris matched with the Geiger Müller counter.

### 3.5 Feasibility for patients

**FIGURE 8:**
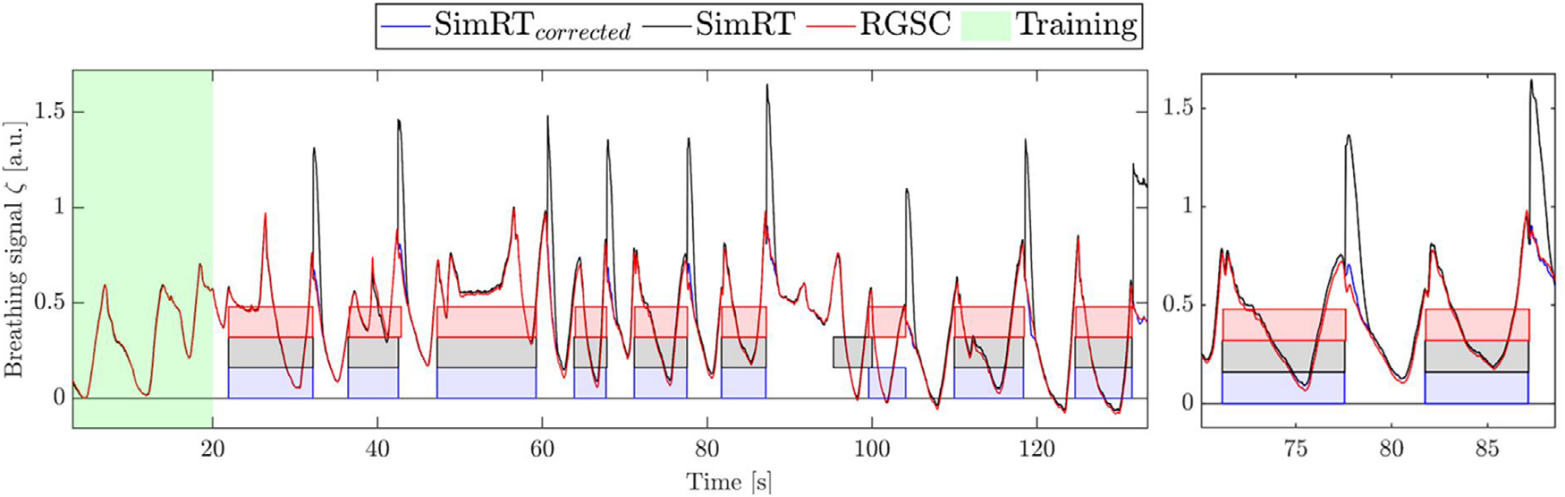
Simulated X‐ray trigger selection in i4DCT in a representative patient case, displaying the entire curve on the left and a zoomed region on the right. The differently colored transparent fields show the simulated X‐ray triggers for the different surrogate systems RGSC, SimRT, and SimRT_corrected_. SimRT_corrected_ and RGSC demonstrated good agreement, whereas SimRT exhibits notable discrepancies, particularly during X‐ray‐off periods where peak artifacts occur.

Patient measurements (see Figure 8) were conducted to validate the phantom results within the clinical context. For the training period, we evaluated RGSC, SimRT, and SimRT_corrected_. The mean BR was 10.59 ± 2.43 BPM for RGSC and 10.58 ± 2.36 for SimRT and SimRT_corrected_. Additionally, the STD of the AMP was 31.34 % for RGSC and 34.55 % for SimRT and SimRT_corrected_.

Analyzing the entire breathing curves, the mean BR was 11.54 ± 4.8 BPM for RGSC, 12.05 ± 5.51 BPM for SimRT_corrected_, and 11.62 ± 4.6 BPM for SimRT. Additionally, the AMP STD was 27.45 % for RGSC, 30.47 % for SimRT_corrected_, and 48.73 % for SimRT. These metrics indicate that the breathing curves of SimRT_corrected_ and RGSC are relatively comparable, though uncorrected SimRT showed highest STD in AMP, likely due to the peak artifacts. The average difference in X‐ray trigger times between RGSC simulated and SimRT was 58.8 ± 85.3 ms. Similarly, for the simulated RGSC versus SimRT_corrected_, the average difference was 68.2 ± 81.8 ms. Regarding X‐ray‐on times, the duration for RGSC was 7.41 ± 2.50 s, for SimRT the duration was 7.43 ± 2.48 s, and lastly, for SimRT_corrected_ the duration was 7.41 ± 2.50 s. Another interesting finding for the case shown in Figure 8 is that SimRT's X‐ray trigger was different at the the 100 s mark of scanning, which was earlier than the counterparts SimRT_corrected_ and RGSC, potentially because of a different X‐ray trigger constraint due to an updated RBC in phase space, due to the peak artifacts.

## 4 DISCUSSION

In this paper, we investigated if a surface monitoring camera system could be modified to offer an eligible alternative to the clinically used IR system. A second IR camera was used as an independent reference. We introduced a technique to address breathing curve artifacts of a surface monitoring system in 4DCT sequence scanning. Although this method has limitations, such as being reliant on the latency from the X‐ray‐on to X‐ray‐off sent from the scanner, it has proven effective in both phantom and patient data. We evaluated *breathing curve quality* and *automatic parameter selection* before scanning for the different surrogate systems in a representative set of phantom measurements. For the *intelligent X‐ray trigger selection*, we measured and simulated X‐ray‐on and X‐ray‐off trigger conditions. Furthermore, assessed the *optimized binning placement* for the reconstruction and its impact on image quality. Lastly, we *simulate latency effects* on reconstructed images and *measured latencies* of different surrogate systems.

In the *breathing curve quality* evaluation, we found that all surrogate systems displayed strong consistency during the training period, as indicated by minimal variation in BR and AMP metrics. For the entire breathing curves, we found that, in comparison to the other surrogate systems for the regular sin‐case and the irregular cos^6^‐case, uncorrected SimRT showed differences in BR and AMP deviation due to peak artifacts, in contrast to the corrected SimRT curves. These findings underscore the effectiveness of the motion corrections applied to SimRT data, and demonstrate the feasibility of using SimRT in this new setting with only relatively minor changes.

The breathing curves from the different surrogate systems showed excellent agreement before irradiation and table movement (see Figure 4). Therefore, even without any corrections applied, we do not expect any differences in the *automatic protocol selection* of Fast 4D for the different surrogate systems.

We found *intelligent X‐ray trigger selection* consistent in our phantom measurements, even for uncorrected curves with minor trigger differences of less than 100 ms (see Figure 4) and well matching X‐ray‐on duration times. The trigger differences were small compared to the mean X‐ray‐on duration times. Extended exposure could result in additional radiation doses, but negligible extra imaging dose is expected when considering very short time intervals using radiation sources tailored for imaging. 4DCT scans still exhibit a high dose compared to other scan protocols. In particular, if multiple follow up scans are done doses can reach concerning levels, which prompted the exploration of dose reduction strategies.^25^ A raised concern revolves around intelligent X‐ray‐on trigger selection for the uncorrected peak artifacts originating from SimRT, which also induces these peak artifacts in the phase space (see Appendix Figure B/C), potentially influencing the updated RBC in phase space, which may impact phase space X‐ray trigger constraints. We are working with the vendor to improve this, as this functionality was not taken into account for the current clinical use.

In this context, the quality of the breathing curves can impact the RBC, which plays a vital role in the *optimized binning placement* and thus the binning placement when reconstructing multiple phases, whether AMP‐ or phase‐based reconstruction is chosen (see Figure 5). AMP‐based reconstruction primarily showed differences during inhalation for the uncorrected surface monitoring system. However, it is less affected by curve quality compared to phase‐based reconstruction. It demonstrated good agreement for COM measurements with deviations of less than 0.5 mm from the independent Polaris reference images, even when using uncorrected SimRT curves. In contrast, phase‐based reconstruction is highly sensitive to the peak artifacts produced by the uncorrected SimRT system, resulting in delayed binning placement and consequently affecting tumor COM positions (see Figure 6).

Results from *latency measurements* revealed that the surface monitoring system SimRT lagged by approximately 45 ms compared to the clinical surrogate system RGSC. This comparison was based on offline .vxp log files that received a TTL‐in impulse from the scanner. Around 80 ms after the TTL‐in impulse was recorded in the offline files, the scanner encoded the X‐ray‐on signal into the 4DCT raw data, which is used for image reconstruction. An additional ∼20 ms later, the Polaris system coupled with the GM10 detected the X‐ray signal. A timeline of the different X‐ray events and signals is provided in the Appendix Figure D.

*Latency simulations* showed that increased latency negatively impacted image quality (see Table 2). These findings underscore the importance of considering latency differences when exchanging breathing signals for retrospective reconstruction.

The RGSC camera system exhibits native latency, resulting in discrepancies between accurate patient breathing data and 4DCT raw data. However, this latency is not critical for the intelligent X‐ray trigger selection. A latency correction method could address issues in image sorting for reconstruction. This method involves shifting the breathing curves to match the patient's breathing before reconstruction, with knowledge of the surrogate system latency. Therefore, while it is a potential error source, it could be corrected, leading to image quality improvements in the current clinical setup with the RGSC and in a setup where surrogate systems would be exchanged. This statement remains valid because binning placement for 4D reconstruction in i4DCT occurs exclusively between the first and second maximum of a breathing curve used for image sorting.

With the therapeutic application of doses to breathing motion affected tumors, two primary strategies are commonly employed to manage respiratory motion during free breathing irradiation: the internal target volume (ITV) approach and the mid‐ventilation (MidV) concept. In our clinic, we employ AMP‐based reconstruction for its reduced vulnerability to artifacts.^26^ Subsequently, we integrate the contouring of gross tumor volumes (GTV) across 10 4DCT phases into a unified ITV to encompass all potential tumor locations. The MidV method treats breathing motion like a random error. It combines this error with other random margins when calculating the PTV margin.^27^ These methods prove resilient against latency‐related issues affecting individual 4DCT phases.

On the other hand, gated RT requires precise phase timing during imaging, thus vulnerable to any latency related issues. Integrating daily 4D dose reconstruction into clinical workflows remains challenging due to limitations in CT data acquisition and the reliance on CT data chosen for all calculations.^28^ To address this, treatment planning 4DCT can be used to generate vector fields from MidV to motion phases. Additionally, daily 4DCTs can be created using imaging data from patient positioning, such as CBCT, and motion surrogates like skin contour, aiding in identifying intra‐fractional motion.^28^, ^29^

While the initial latency results are promising, further studies in real‐world settings, rather than simulations, would provide valuable insights into the latency's impact on image quality. One limitation of the study is that only raw data by steering the scanner with the RGSC was recorded. Thus, direct latency measurements, such as prospectively steering the scanner with different surrogate signals, could be conducted. However, addressing any breathing curve issues beforehand is essential to prevent overlapping influences on the analysis, which could otherwise complicate interpretation.

The integration of surface‐guided surrogates in sequence scanning for GE scanners, as described by Spadea et al. involving GateCT (a predecessor to SimRT), shares certain similarities with the sequence scan mode of i4DCT.^30^ The study reported that couch motion was accounted for via software based on specific CT parameters, similar to the table correction applied in our study. However, it also emphasized that respiratory waveforms were unreliable during couch motion, highlighting a key limitation of the study. While both approaches investigated the use of surface‐guided surrogates in sequence scanning, i4DCT represents a more advanced approach by utilizing real‐time surrogate information and intelligent X‐ray trigger selection, placing greater demands on the surrogate system.

Currently, it is not feasible to directly control the scanner in real‐time with a surrogate system other than the RGSC IR camera and the Anzai breathing belt. Many surrogates aren't equipped to actively control the scanner and provide real‐time data at the required speed. Although SimRT's current solution does not support streaming real‐time data, this aligns with its original design, as it was not intended for such functionality in the clinical version. For the Polaris setup, however, a custom solution would need to be developed to enable this capability. Additionally, unlike the conventional 4DCT approach, which already has a working solution for swapping surrogate systems, the i4DCT lacks an open interface for real‐time data exchange.

This study primarily addressed technical feasibility, with a thorough image quality comparison in the phantom setting still to be conducted. As part of the phantom study, minimizing the influence of partial volume effects and assessing potential artifacts from varying breathing curve qualities would require reconstructing as many phases as possible (e.g., 20 phases) with a thin slice thickness (e.g., 1 mm).

Our study found satisfactory breathing curve quality results after correcting for the alternative surrogates Polaris and SimRT compared to our clinical system RGSC. Despite this, RGSC remains the reliable workhorse for i4DCT imaging. However, using the Polaris with a combined multi‐tracking approach could mitigate potential table sag issues, but this is not planned for a clinical release. Adopting SimRT may be attractive due to its seamless workflow integration from the imaging site to the accelerator site, along with offering a markerless solution. Therefore, we are actively working with the vendor to implement this new functionality in a future release of SimRT.

## 5 CONCLUSION

The technical feasibility of applying a surface monitoring system to breathing‐adapted i4DCT was demonstrated. Although not originally designed for this specific application, the surface monitoring system, after undergoing modifications, achieved satisfactory alignment with other systems in generating breathing‐adapted 4DCT scans. The recommended modifications included correcting the breathing curves for table motion. Without these modifications, discrepancies were identified in the breathing signals derived from surface monitoring, primarily due to table motion, which negatively impacted reconstruction binning placement and image quality. Initial latency analysis indicates that surface monitoring lags behind the clinical system in signal creation in its current version. Further research is recommended to investigate the effect of latency on image quality in experimental settings to determine if this effect is negligible.

## AUTHOR CONTRIBUTIONS

All authors contributed significantly to the performed work and approved its final version to be published. Niklas Lackner (NL), Andre Karius (AK), Rainer Fietkau (RF), Christoph Bert (CB), Juliane Szkitsak (JS), Conceptualization: NL, AK, CB, JS. Methodology: NL, AK, CB, JS. Software/Tools Development: NL, AK. Data collection & analysis: NL. Writing—Original Draft Preparation: NL. Writing—Review & Editing: NL, AK, CB, RF, JS. Supervision: CB, JS.

## CONFLICT OF INTEREST STATEMENT

The Universitätsklinikum Erlangen and the Department of Radiation Oncology have institutional research grants with Siemens Healthineers AG and VisionRT that are not directly related to the present work.

## Supporting information



Supporting Information

## Data Availability

Data are provided by the corresponding author upon reasonable request.
