# Supplementary material for: Exploring surface‐guided systems for intelligent breathing‐adapted four‐dimensional computed tomography: A comparison to infrared‐based reflective marker systems
Source: J Appl Clin Med Phys. 2025 Feb 24;26(5):e70054. doi: 10.1002/acm2.70054 (PMC12059290; doi:10.1002/acm2.70054)
Supplement: Supplementary file 1 — Supporting Information [file ACM2-26-e70054-s001.docx]

# Appendix

**SimRT peak-artifact creation due to patch propagation**

This section discusses the SimRT peak artifact behavior specific to i4DCT scanning, which differs from most other 4DCT acquisitions. i4DCT employs axial (sequence) CT scanning, where the scanning process (X-ray-on) and table movement are disconnected. The table moves with a consistent increment between scans: 0.9x64x0.6 mm (34.56 mm total). The table movement occurs with a delay after switching from X-ray-on to X-ray-off.

Understanding artifact creation begins with SimRT capturing the surface of an object, followed by patch selection. The breathing data is then acquired by calculating real-time position deviations of a surface region using a rigid-body transformation between the reference and the current surface. In i4DCT, when the X-ray transitions from on to off, the table starts moving, causing the patch to jump to a new position 34.56 mm deeper into the scanner (must be set as correction value in SimRT). This transition can cause a peak artifact (Figure A-c), especially on sloped surfaces (Figure A-b), due to the patch's advanced positioning compared to the table's movement. On a surface without a slope relative to the CT scanner, no peak occurs. The peak height can be used to derive a camera angle-dependent factor. This scaling factor in SimRT is likely necessary for amplitude stability as the patch travels deeper into the CT scanner.

**
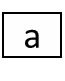

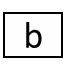
**
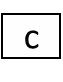

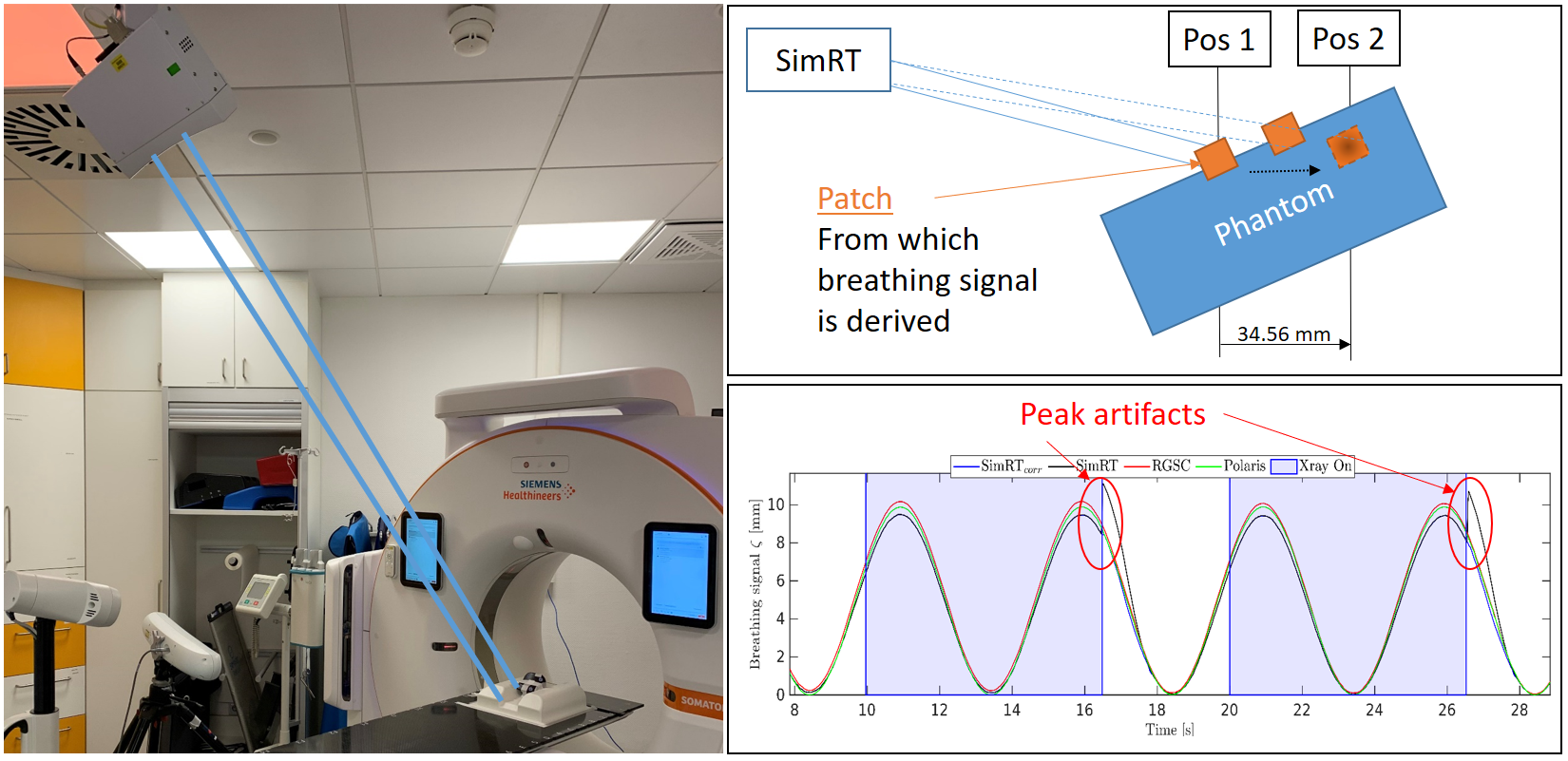


***Figure A.*** *a) Experimental setup displaying SimRT's camera angle. b) Sketch illustrating patch propagation using SimRT in i4DCT scan mode. c) Generic breathing signal for a CIRS dynamic thorax acquisition with the white thorax test phantom on top. The red highlighted regions indicate peak artifacts, with varying heights depending on the camera angles.*

**Reference Breathing Curves in Phase Space**

Here, the breathing curves used are shown in phase space representations (Figure B), which serve as a basis for dynamically adjusting X-ray-on selection. This adjustment is achieved through phase space parameter constraints (Figure C) that are defined on a reference breathing curve for each table position. By synchronizing with the patient's ongoing breathing frequency, this method ensures complete cycle acquisition.

**
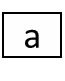

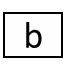
**
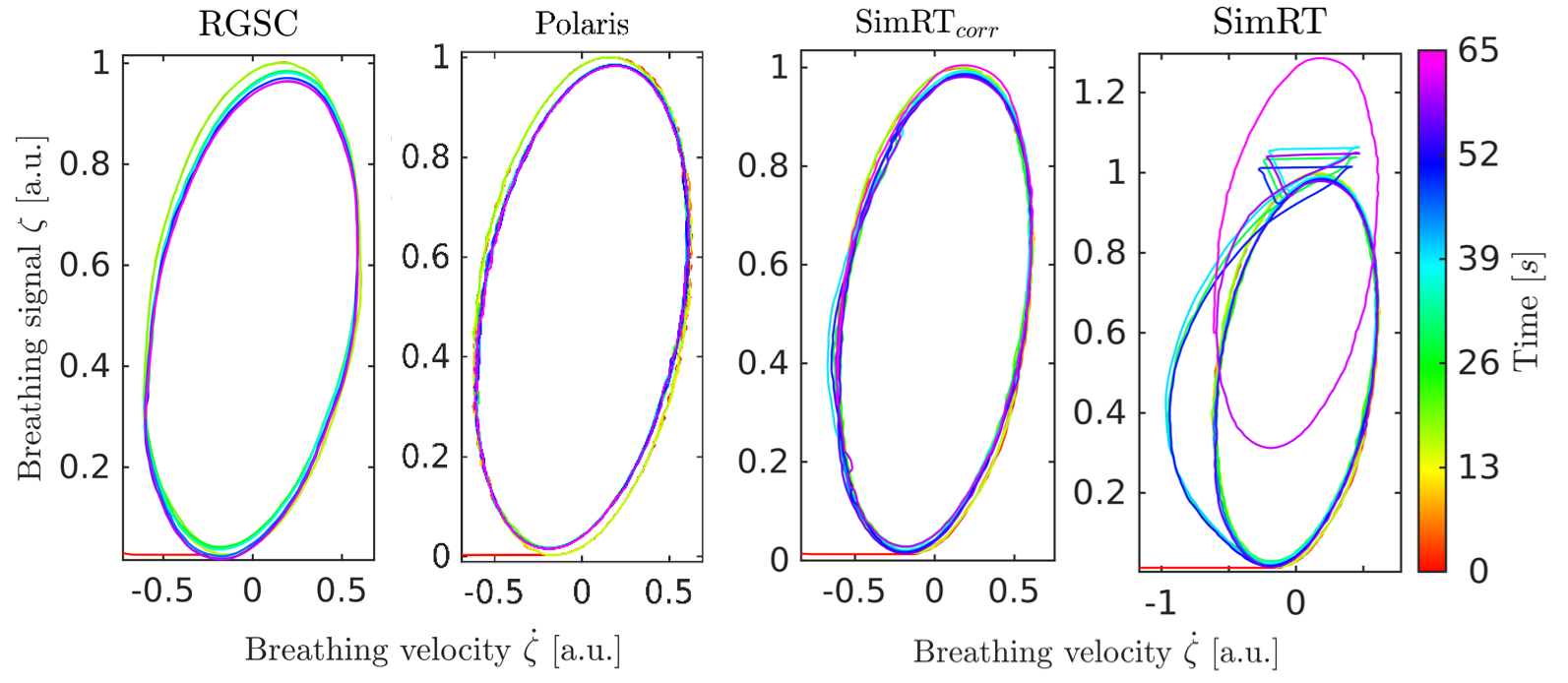

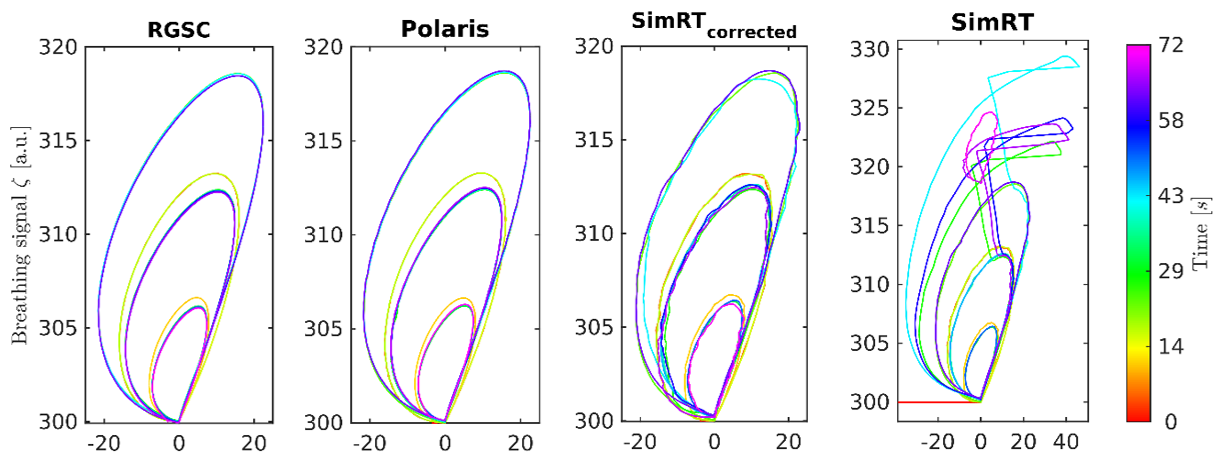


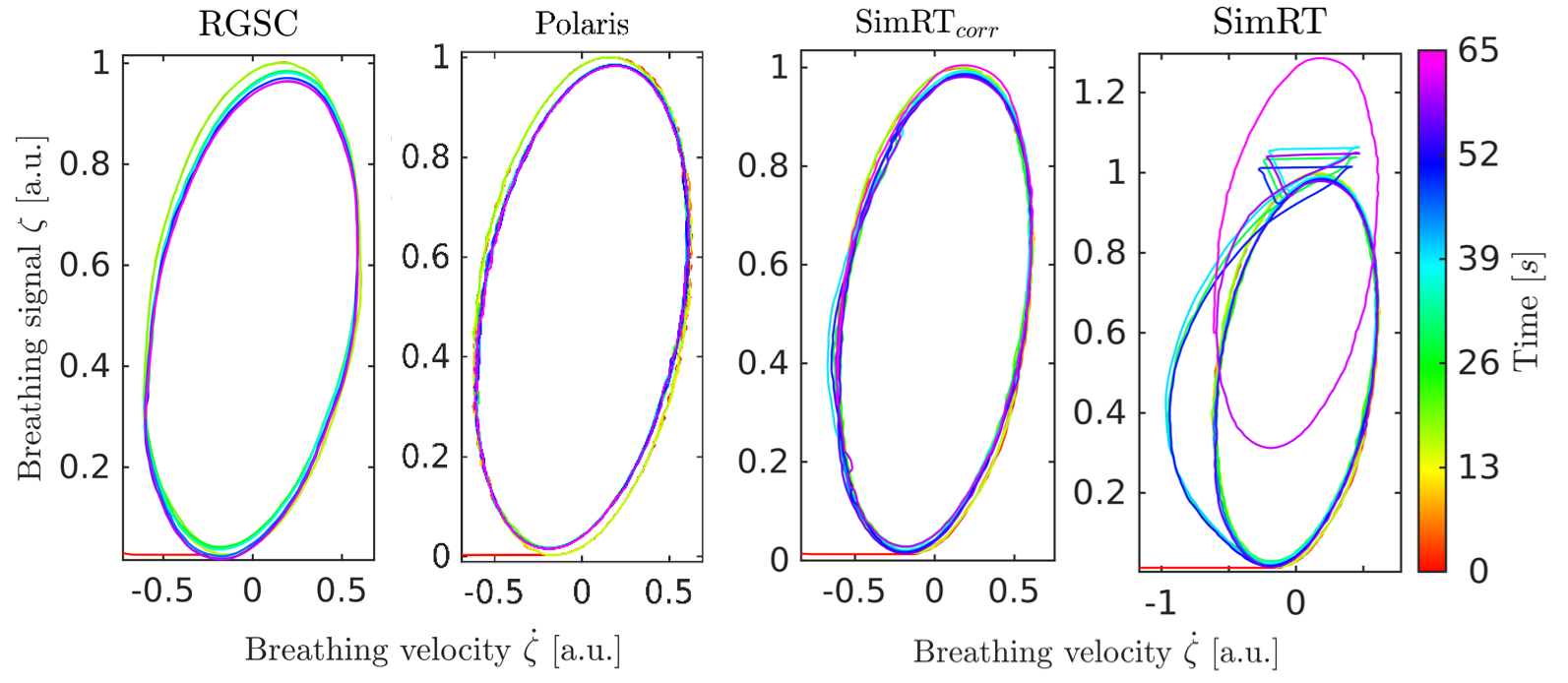


***Figure B.*** *The breathing curves in phase space representations of the different surrogates are shown for a) a regular sin-case and b) an irregular cos^6^-case. The phase space curves of SimRT_corrected_, RGSC, and Polaris show good alignment, while SimRT displays significant deviations, noticeable as spike artifacts. In extreme cases, these artifacts could result in a shifted reference breathing curve in phase space, leading to potential different X-ray-on/-off trigger constraints.*

*
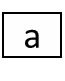

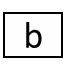
*
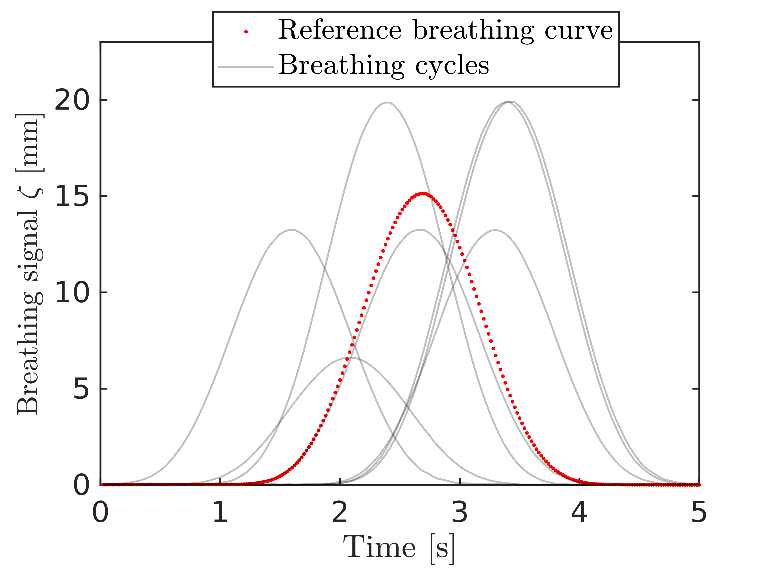

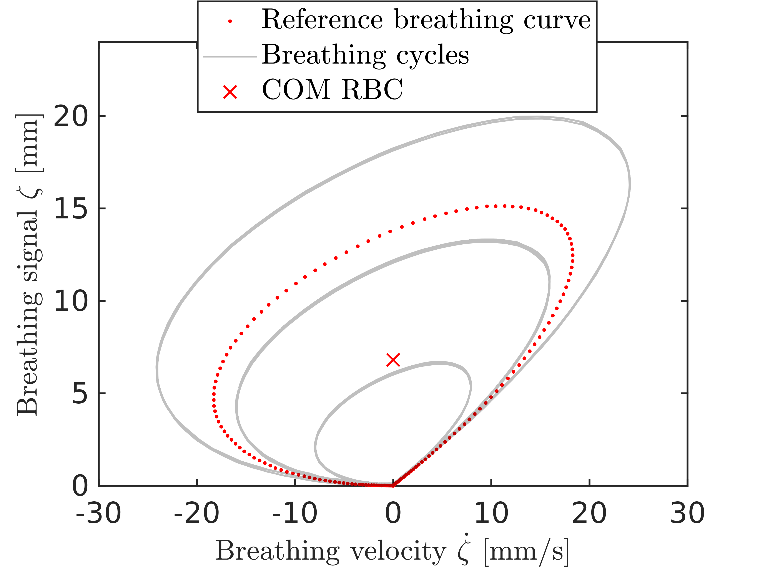


***Figure C.*** *Exemplary irregular motion phantom breathing curves illustrating the terms used in the i4DCT algorithm.
a) Reference breathing curve (RBC) with corresponding breathing cycles. b) Phase space representation of the reference breathing curve, and corresponding breathing cycles and the center of mass (COM) for the RBC in the phase space - only the first few cycles are shown for clarity.*

**TTL-in and X-ray-on Timeline**


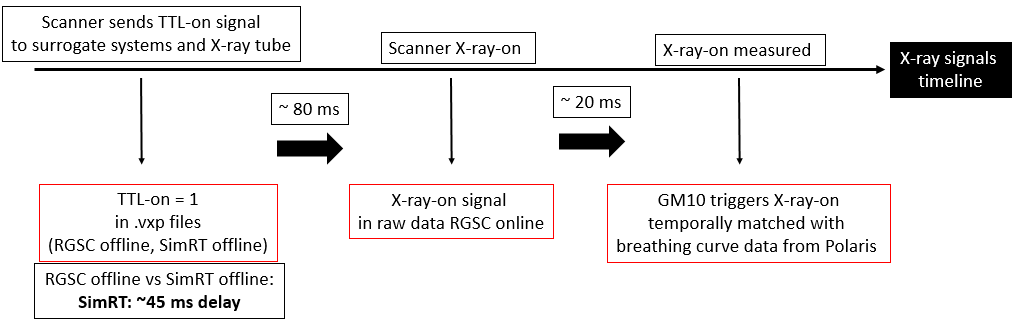


***Figure D.*** *Timeline of the different X-ray signals for the different systems. The timing sequence starts with the scanner sending a TTL signal to the surrogates (RGSC offline, SimRT offline) and to the X-ray tube. Roughly 80 ms after the RGSC offline signal captures the X-ray event, the scanner activates the X-ray, which is then recorded in the RGSC online signal within the 4DCT raw data. Approximately 20 ms after an X-ray-on signal is noted in the RGSC online data, the GM10 detects the X-ray, synchronized with the Polaris breathing signal.*
